# Risk Factors for High-Arched Palate and Posterior Crossbite at the Age of 5 in Children Born Very Preterm: EPIPAGE-2 Cohort Study

**DOI:** 10.3389/fped.2022.784911

**Published:** 2022-04-15

**Authors:** Sandra Herrera, Véronique Pierrat, Monique Kaminski, Valérie Benhammou, Laetitia Marchand-Martin, Andrei S. Morgan, Elvire Le Norcy, Pierre-Yves Ancel, Alice Germa

**Affiliations:** ^1^Université de Paris, Epidemiology and Statistics Research Center/CRESS, INSERM, INRA, Paris, France; ^2^CHU Lille, Department of Neonatal Medicine, Jeanne de Flandre Hospital, Lille, France; ^3^Elizabeth Garrett Anderson Institute for Women's Health, University College London, London, United Kingdom; ^4^Department of Neonatal Medicine, Maternité Port-Royal, Paris, France; ^5^Université de Paris, EA2496, Montrouge, France; ^6^Clinical Research Unit, Centre for Clinical Investigation P1419, Cochin Broca Hôtel-Dieu Hospital, Paris, France; ^7^Department of Odontology, APHP, Charles Foix Hospital, Ivry-sur-Seine, France

**Keywords:** cohort study, very preterm, high-arched palate, posterior crossbite, non-nutritive sucking habits, cerebral palsy

## Abstract

**Introduction:**

Children born very preterm have an immature sucking reflex at birth and are exposed to neonatal care that can impede proper palate growth.

**Objectives:**

We aimed to describe the frequency of high-arched palate and posterior crossbite at the age of 5 in children born very preterm and to identify their respective risk factors.

**Methods:**

Our study was based on the data from EPIPAGE-2, a French national prospective cohort study, and included 2,594 children born between 24- and 31-week gestation. Outcomes were high-arched palate and posterior crossbite. Multivariable models estimated by generalized estimation equations with multiple imputation were used to study the association between the potential risk factors studied and each outcome.

**Results:**

Overall, 8% of children born very preterm had a high-arched palate and 15% posterior crossbite. The odds of high-arched palate were increased for children with low gestational age (24–29 vs. 30–31 weeks of gestation) [adjusted odds ratio (aOR) 1.76, 95% confidence interval (CI) 1.17, 2.66], thumb-sucking habits at the age of 2 (aOR 1.53, 95% CI 1.03, 2.28), and cerebral palsy (aOR 2.18, 95% CI 1.28, 3.69). The odds of posterior crossbite were increased for children with pacifier-sucking habits at the age of 2 (aOR 1.75, 95% CI 1.30, 2.36).

**Conclusions:**

Among very preterm children, low gestational age and cerebral palsy are the specific risk factors for a high-arched palate. High-arched palate and posterior crossbite share non-nutritive sucking habits as a common risk factor. The oro-facial growth of these children should be monitored.

## Introduction

Each year, 2.4 million babies are born very preterm worldwide (before 32-week gestation) (1). Children born very preterm are exposed to a wide range of major and minor health problems and impairments, which includes maxillofacial growth anomalies such as high-arched palate and posterior crossbite (2).

Both are maxillofacial growth anomalies occurring in the transverse plane and seem very related to each other. A high-arched palate (or high-vaulted palate) is characterized as an unusually high and narrow palate and thus may result in higher risk for malocclusion. Posterior crossbite is a transverse malocclusion most often associated with a narrow upper dental arch (the palatal cusps of the upper teeth do not fit in the central fossae of the lower teeth as expected) (3). Posterior crossbite may have functional consequences (difficulties in chewing and phonation), may, in some cases, lead to pain (in chewing muscles and in the temporomandibular joint), and may affect esthetics or generate psychological issues that may negatively affect emotional and social well-being (3–6). In addition, high-arched palate is often associated with decreased nasal airway volume, which predisposes the child to sleep disordered breathing (such as obstructive sleep apnea) (7, 8).

The frequency of high-arched palate has not been established owing to the lack of a standard definition (9, 10). Posterior crossbite has been estimated to occur in at least 17% of children born preterm (11) (13% of children born full-term) (12).

The transverse growth of the palate is enhanced by the pressure of the tongue against the palate. Insufficient transverse growth may occur if the tongue is not sufficiently competent (e.g., with neuromotor dysfunctions) (13) or if it remains in a low position (e.g., with a pacifier habit). Prolonged non-nutritive sucking habits (NNSHs, i.e., sucking a pacifier or thumb) are the main risk factors for both high-arched palate and posterior crossbite in children in the general population (14–17). Pacifier sucking is common and even recommended in infants born very preterm during neonatal hospitalization to support maturation of oro-facial motor function and to minimize pain (18–20). The frequency of NNSHs during infancy seems higher in infants born preterm than those born full-term (21).

Moreover, infants born very preterm may be exposed to intubation and tube feeding enhancing low tongue positioning. Consequently, identifying among factors linked to preterm birth those associated with maxillofacial growth anomalies may contribute to early diagnosis, thus facilitating timely interceptive orthodontic treatment (22).

Our work is based on EPIPAGE-2 data. EPIPAGE-2 is a French nationwide population-based cohort study designed to follow very preterm children born in 2011 during their first 12 years of life (23). The objectives of this current work, which is a part of the comprehensive 5-year medical and neurodevelopmental follow-up in the EPIPAGE-2 cohort study, were to describe the frequency of high-arched palate and posterior crossbite at the age of 5 and to identify the respective risk factors including neonatal characteristics and care, NNSHs at age 2, and cerebral palsy (CP) at the age of 5.

## Methods

### Population

Children included in this study were the part of the EPIPAGE-2 national population-based cohort study (described elsewhere) (23). Briefly, children were recruited in 2011, during 8 months for those born between 24 and 26 completed weeks of gestation and during 6 months for those born between 27 and 31 weeks of gestation. Survivors were enrolled for longitudinal follow-up and were included in the study at the age of 2 corrected age and age 5$\frac{1}{2}$ (hereafter age 5) if their parents consented. In this study, we included children with complete data on their NNSHs (pacifier or thumb/fingers) at the age of 2.

### Data Collection

EPIPAGE-2 data were collected by the use of standardized questionnaires during the neonatal period and at the ages of 2 and 5. Neonatal questionnaires included demographic, social and delivery data, infant's condition at birth, neonatal complications, and care received in the neonatal intensive care unit (NICU). Pacifier and thumb-sucking habits were collected by the use of a self-administered questionnaire completed by the parents at the age of 2. At the age of 5, children were examined by pediatricians specifically trained for the study. This examination included a neurodevelopmental evaluation with the diagnosis of CP and a clinical oral assessment for high-arched palate and posterior crossbite.

### Main Outcomes

The primary outcomes were high-arched palate and posterior crossbite (No/Yes) at the age of 5. High-arched palate was defined as deep and narrow. Posterior crossbite was defined as at least one mandibular molar cusp positioned buccal to the maxillary cups. Pediatricians were assisted in their assessment of high-arched palate and posterior crossbite by a guidance chart with illustrative drawings and photos (refer to Supplementary Figure S1).

### Potential Risk Factors

The factors studied were selected based on their association with high-arched palate and posterior crossbite shown in the general population (24, 25) or in children born preterm (13, 26–29) and were defined as (1) neonatal characteristics: sex, gestational age (defined as the best obstetric estimate, combining the last menstrual period and the first-trimester ultrasonography assessment), small-for-gestational-age (defined as birth weight less than the 10th percentile according to the gestational age and sex based on French EPOPé intrauterine growth curves) (30), and severe neonatal morbidities (31) (including severe bronchopulmonary dysplasia, severe necrotizing enterocolitis, severe retinopathy, or severe cerebral abnormalities defined as intraventricular hemorrhage grade III/IV or cystic periventricular leukomalacia; definitions are provided in the note to Appendix 1); (2) neonatal care practices: duration of intubation (none or <24 h, 24 h−28 days, >28 days), feeding by nasogastric tube at 36 weeks, oral stimulation, and breastfeeding at discharge; (3) pacifier and thumb-sucking habits at the age of 2; and (4) CP at the age of 5 defined according to the criteria of the Surveillance of Cerebral Palsy in Europe (32) network.

### Statistical Analysis

Because the population was selected based on the children who survived to the age of 5 with complete data for NNSHs at the age of 2, we described perinatal and sociodemographic characteristics of children with complete data for NNSHs at the age 2 but with missing data for the outcomes at the age of 5 and those with missing data for NNSHs at the age of 2.

Frequencies of high-arched palate and posterior crossbite are described according to all the studied factors. All percentages and odds ratios (ORs) were weighted to account for differences in the sampling process between gestational age groups.

The adjusted analyses involved multivariable logistic regression models to identify the factors associated with high-arched palate and posterior crossbite, which includes neonatal factors (i.e., sex, gestational age, and small-for-gestational-age), neonatal morbidities and care practices (i.e., duration of intubation, oral stimulation, and breastfeeding at discharge), pacifier and thumb-sucking habits at the age of 2, and CP at the age of 5.

Intubation may be considered a marker of poor health, but endotracheal tubes also potentially exert pressure on the palate. Because of its relation with the palate area, duration of intubation was used in the final model rather than severe neonatal morbidities or feeding by nasogastric tube at 36 weeks, both being closely linked to duration of intubation.

Because twins and triplets share pregnancy and family characteristics, we used generalized estimation equations to take into account intra-family correlations (33). Results are reported as adjusted ORs (aORs) with 95% confidence intervals (95% Cls).

The analyses involved using imputed datasets to limit the possible impact of both lost to follow-up and missing data on the outcomes and covariates (34–36). The imputed datasets using chained equations were created using variables that potentially predicted non-response on the outcomes. Missing data were 36% of the outcome measures, and among covariates, 0% of neonatal factors, fewer than 6% of neonatal morbidities and care practices, and 20% of CP. In total, 50 independent imputed datasets were generated. Estimates were pooled according to the Rubin's rule (37). Further imputation details are provided in Supplementary Table S1. Statistical analyses were performed with *R* v3.6.3.

A total of two sensitivity analyses were performed, one of complete cases and the other of all children surviving at the age of 2 including those with missing data for NNSHs at the age of 2.

## Results

### Study Population

**Figure 1:**
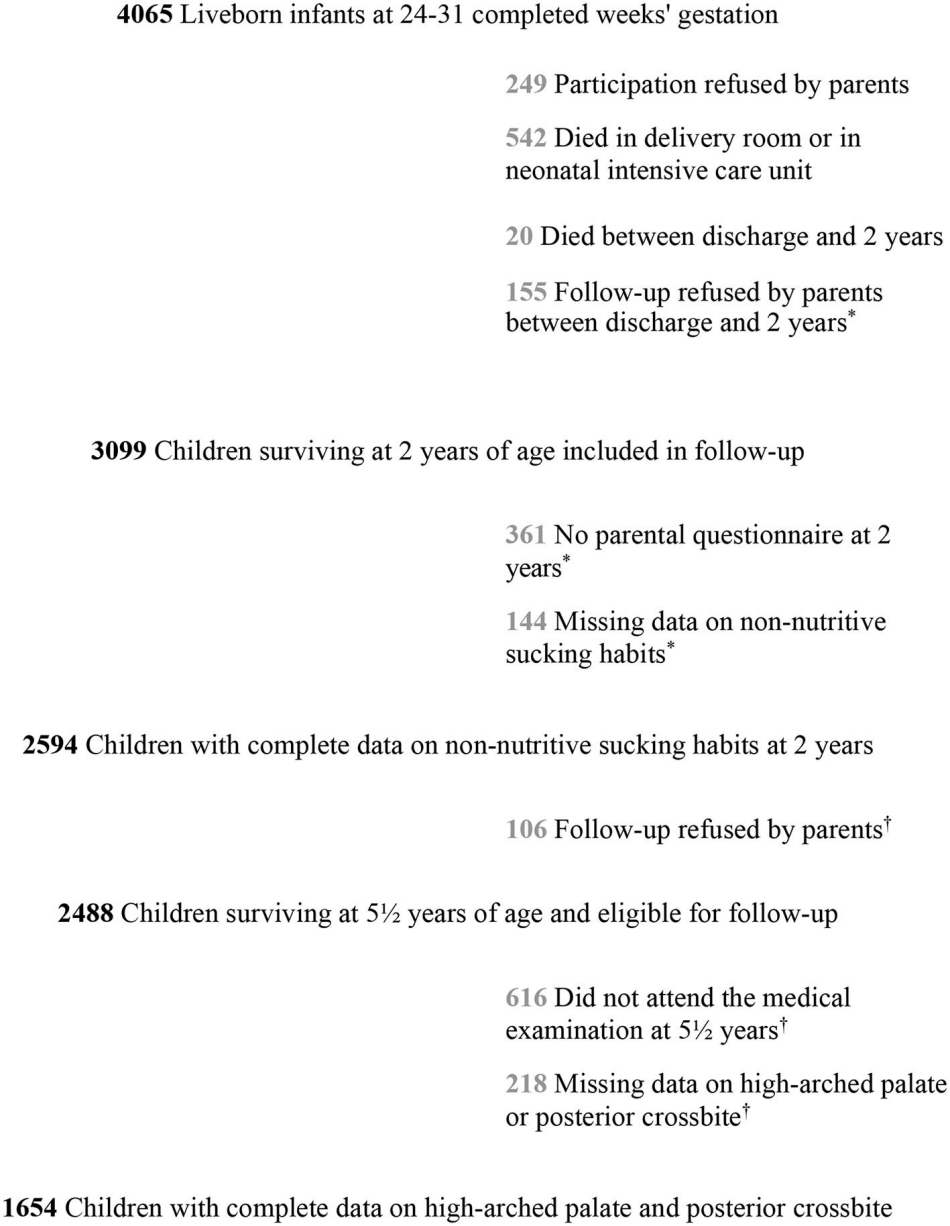
Flow chart of the study population: EPIPAGE-2 cohort at 5$\frac{1}{2}$ years. ^*^Considered “missing data on non-nutritive sucking habits at 2 years' corrected age” = **660 children**. Considered “missing data at 5$\frac{1}{2}$ years” = **940 children**.

Among the 2,594 children alive at the age of 2 with the complete data on NNSHs, 2,488 were eligible for follow-up at the age of 5, and 1,654 had a medical examination with complete data regarding high-arched palate and posterior crossbite (Figure 1).

As compared to children included in the analysis, those with missing data at the age of 5 and those with missing data on NNSHs were more often born to mothers born outside France or to parents with low socioeconomic status and with 2 or more older siblings and were less frequently breastfed at discharge (Table 1).

**Table 1:** Maternal, neonatal, and follow-up characteristics of the study population and those with missing data.

	**Study**		***NNSHs available but with***		***Missing data for***	
	**population**		***missing data at 5***$\frac{1}{2}$ ***years***		***NNSHs at 2 years***	
	***N***	**%^a^**	***n***	**%^a^**	***n***	**%^a^**
Total	**1,654**	100	**940**	100	**660**	100
***Maternal characteristics***						
Country of birth						
France	1,329	80.4	693	74.9	434	68.2
Other	323	19.6	239	25.1	200	31.8
**Parents' socioeconomic status** ^b^						
Professional	413	26.0	172	19.1	71	12.0
Intermediate	395	24.8	164	18.5	72	12.3
Administrative, public service, self-employed, students	403	25.7	263	29.2	174	29.5
Shop assistants, service workers	203	12.5	140	15.6	106	18.0
Manual workers	150	9.4	121	13.5	119	20.0
Unknown	22	1.6	38	4.1	49	8.2
**Parity**						
0	952	57.9	495	52.8	299	46.5
1	386	23.7	214	23.2	160	24.5
≥2	299	18.4	226	24.0	187	29.0
**Type of pregnancy**						
Single	1,101	66.6	631	67.0	468	71.0
Multiple	553	33.4	309	33.0	192	29.0
***Neonatal characteristics and care practices***						
**Sex**						
Boys	846	51.2	502	53.5	353	53.5
Girls	808	48.8	438	46.5	307	46.5
**Gestational age (weeks)**						
24–26	261	12.2	161	13.3	122	13.0
27–29	638	40.2	348	38.7	235	39.2
30–31	755	47.6	431	48.0	303	47.8
**Small-for-gestational age** ^c^						
No	1,064	63.7	620	65.3	435	65.0
Yes	590	36.3	320	34.7	225	35.0
**Severe neonatal morbidities** ^d^						
No	1,329	85.0	749	84.6	513	83.5
Yes	254	15.0	147	15.4	109	16.5
**Intubation**						
<24 h	509	32.1	302	33.7	206	33.0
24 h−28 days	1,035	62.5	564	60.0	406	61.7
>28 days	104	5.4	69	6.3	39	5.3
**Feeding by nasogastric tube at 36 weeks**						
No	489	34.1	299	36.3	232	42.4
Yes	981	65.9	548	63.7	330	57.6
**Oral stimulation**						
No	444	28.4	274	31.0	190	32.2
Yes	1,131	71.6	606	69.0	410	67.8
**Breastfeeding at discharge**						
No	901	57.1	603	66.8	438	71.3
Yes	669	42.9	296	33.2	173	28.7
***Follow-up characteristics***						
**Pacifier-sucking at 2 years**						
No	720	43.5	430	45.3		
Yes	934	56.5	510	54.7		
**Thumb-sucking at 2 years**						
No	1,282	77.5	732	78.2		
Yes	372	22.5	208	21.8		
**Cerebral palsy at 5**$\frac{1}{2}$ **years**						
No	1,551	94.3				
Yes	96	5.7				

*NNSHs, non-nutritive sucking habits*.

a *Percentages weighted to account for differences in the sampling process between gestational age groups*.

b *Defined as the highest occupational status of the mother and father or occupation of mother only if living alone*.

c *Defined as birth weight less than the 10th centile for gestational age and sex based on French EPOPé intrauterine growth curves (Ego 2016)*.

d *Defined as severe bronchopulmonary dysplasia or necrotizing enterocolitis stage 2–3, severe retinopathy of prematurity stage >3 or any of the following severe cerebral abnormalities on cranial ultrasonography: intraventricular hemorrhage grade III/IV or cystic periventricular leukomalacia*.

### Frequency of High-Arched Palate and Posterior Crossbite

Among the 1,654 children with available information, 7.5% (95% CI 6.0–9.0) had a high-arched palate and 15.0% (95% CI 13.3–16.8) had a posterior crossbite (Table 2). After multiple imputation, the frequency of high-arched palate was 8.0% (95% CI 7.0–10.0), that of posterior crossbite was 15.0% (95% CI 13.7–16.5), that of anterior crossbite was 12% (95% CI 11.1–13.7), that of bilateral was crossbite 3% (95% CI 2.0–4.0), and that of complete crossbite (anterior crossbite and bilateral crossbite) was 1% (data not shown).

**Table 2:** Frequency of high-arched palate and posterior crossbite at 5$\frac{1}{2}$ years by neonatal characteristics, non-nutritive sucking habits (NNSHs) at 2 years, and cerebral palsy at 5$\frac{1}{2}$ years: complete cases (*n* = 1,654).

		**High-arched palate**			**Posterior crossbite**		
	***N***	***n***	**%^a^**	***P^b^***	***n***	**%^a^**	***P^b^***
Total	**1,654**	125	7.5		248	15.0	
**Type of pregnancy**							
Single	1,101	91	8.2	0.19	158	14.3	0.35
Multiple	553	34	6.3		90	16.2	
**Sex**							
Boys	846	64	7.6	0.99	129	15.2	0.78
Girls	808	61	7.5		119	14.6	
**Gestational age (weeks)**							
24–26	261	20	7.7	0.004	42	16.1	0.70
27–29	638	64	10.0		99	15.5	
30–31	755	41	5.4		107	14.1	
**Small-for-gestational age** ^c^							
No	1,064	76	7.1	0.36	160	15.0	0.09
Yes	590	49	8.4		88	14.9	
**Severe neonatal morbidities** ^d^							
No	1,329	94	7.0	<0.001	195	14.6	0.03
Yes	254	25	17.0		50	20.1	
**Intubation**							
<24 h	509	27	5.3	0.04	80	15.6	0.84
24 h−28 days	1,035	88	8.5		152	14.6	
>28 days	104	10	10.1		14	14.5	
**Feeding by nasogastric tube at 36 weeks**							
No	489	36	7.2	0.05	79	16.0	0.70
Yes	981	79	8.1		149	15.2	
**Oral stimulation**							
No	444	32	7.2	0.94	56	12.8	0.12
Yes	1,131	85	7.5		182	16.0	
**Breastfeeding at discharge**							
No	901	79	8.8	0.03	147	16.3	0.05
Yes	669	39	5.8		86	12.7	
**Pacifier-sucking at 2 years**							
No	720	50	6.7	0.30	223	15.1	<0.001
Yes	934	75	8.1		25	13.4	
**Thumb-sucking at 2 years**							
No	1,282	86	6.8	0.04	191	15.0	0.90
Yes	372	39	10.2		57	15.2	
**Cerebral palsy at 5**$\frac{1}{2}$ **years**							
No	1,551	109	7.0	<0.001	224	14.4	0.02
Yes	96	15	16.7		22	23.3	

a *Percentages weighted to account for differences in the sampling process between gestational age groups*.

b *Pearson chi-square p-value*.

c *Defined as birth weight less than the 10th centile for gestational age and sex based on the French EPOPé intrauterine growth curves (Ego 2016)*.

d *Defined as severe bronchopulmonary dysplasia or necrotizing enterocolitis stage 2–3 or severe retinopathy of prematurity stage >3 or any of the following severe cerebral abnormalities on cranial ultrasonography: intraventricular hemorrhage grade III/IV or cystic periventricular leukomalacia*.

Table 3 shows the association between the two anomalies. In 70% of cases of high-arched palate, there was no posterior crossbite.

**Table 3:** Association between high-arched palate and posterior crossbite at 5$\frac{1}{2}$ years.

	**Posterior crossbite**			
	***N***	***n***	**%^a^**	***P^b^***
**High-arched palate**				
No	1,529	211	13.8	<0.001
Yes	125	37	29.6	

a *Percentages weighted to account for differences in the sampling process between gestational age groups*.

b *Pearson chi-square p-value*.

### Factors Associated With High-Arched Palate and Posterior Crossbite

Table 2 shows the distribution of high-arched palate and posterior crossbite according to the factors studied. After adjustment (Table 4), the odds of high-arched palate were increased for children with thumb-sucking habits at the age of 2 and children with CP. The odds of high-arched palate were increased with low gestational age (*p* = 0.05) and the lack of breastfeeding at discharge (*p* = 0.08), although not significantly. High-arched palate was not associated with sex, duration of intubation, oral stimulation in a NICU, or pacifier-sucking habits.

**Table 4:** High-arched palate and posterior crossbite at 5$\frac{1}{2}$ years by neonatal characteristics, non-nutritive sucking habits (NNSHs) at 2 years and cerebral palsy at 5$\frac{1}{2}$ years: unadjusted and adjusted odds ratios (ORs), multivariable regression models with generalized estimating equations (GEEs) and multiple imputation (*n* = 2,594).

	**High-arched palate**					**Posterior crossbite**			
	**Unadjusted**		**Adjusted**			**Unadjusted**		**Adjusted**	
	**OR (95% CI)^a^**	***P^b^***	**aOR (95% CI)^c^**	***P^b^***		**OR (95% CI)^a^**	***P^b^***	**aOR (95% CI)^c^**	***P^b^***
	***N*** **= 2,594**		***N*** **= 2,594**			***N*** **= 2,594**		***N*** **= 2,594**	
**Sex**									
Boys	1.00 (Reference)	0.73	1.00 (Reference)	0.57	Boys	1.00 (Reference)	0.81	1.00 (Reference)	0.57
Girls	0.94 (0.67, 1.32)		0.90 (0.63, 1.29)		Girls	0.97 (0.75, 1.25)		0.91 (0.68, 1.24)	
**Gestational age (weeks)**									
24–26	1.56 (0.93, 2.62)	0.01	1.30 (0.69, 2.45)	0.05	24–26	1.18 (0.81, 1.73)	0.30	1.38 (0.84, 2.26)	0.20
27–29	1.94 (1.33, 2.85)		1.76 (1.17, 2.66)		27–29	1.15 (0.85, 1.56)		1.26 (0.88, 1.80)	
30–31	1.00 (Reference)		1.00 (Reference)		30–31	1.00 (Reference)		1.00 (Reference)	
**Small-for-gestational age** ^d^									
No	1.00 (Reference)	0.56	1.00 (Reference)	0.41	No	1.00 (Reference)	0.92	1.00 (Reference)	0.73
Yes	1.11 (0.78, 1.59)		1.16 (0.81, 1.65)		Yes	0.99 (0.75, 1.30)		0.95 (0.73, 1.24)	
**Intubation**									
<24 h	1.00 (Reference)	0.01	1.00 (Reference)	0.34	<24 h	1.00 (Reference)	0.58	1.00 (Reference)	0.13
24 h−28 days	1.67 (1.14, 2.44)		1.31 (0.87, 1.99)		24 h−28 days	0.92 (0.69, 1.22)		0.79 (0.56, 1.13)	
>28 days	2.01 (1.03, 3.94)		1.38 (0.61, 3.14)		>28 days	0.80 (0.44, 1.43)		0.60 (0.28, 1.27)	
**Oral stimulation**									
No	1.00 (Reference)	0.49	1.00 (Reference)	0.41	No	1.00 (Reference)	0.10	1.00 (Reference)	0.09
Yes	0.88 (0.60, 1.27)		0.85 (0.59, 1.24)		Yes	1.29 (0.95, 1.75)		1.31 (0.96, 1.79)	
**Breastfeeding at discharge**									
No	1.00 (Reference)	0.03	1.00 (Reference)	0.08	No	1.00 (Reference)	0.06	1.00 (Reference)	0.09
Yes	0.65 (0.45, 0.96)		0.71 (0.48, 1.04)		Yes	0.76 (0.58, 1.01)		0.79 (0.59, 1.04)	
**Pacifier-sucking at 2 years**									
Non	1.00 (Reference)	0.29	1.00 (Reference)	0.18	Non	1.00 (Reference)	<0.001	1.00 (Reference)	<0.001
Yes	1.22 (0.84, 1.76)		1.27 (0.89, 1.81)		Yes	1.67 (1.28, 2.18)		1.75 (1.30, 2.36)	
**Thumb-sucking at 2 years**									
Non	1.00 (Reference)	0.03	1.00 (Reference)	0.03	Non	1.00 (Reference)	0.83	1.00 (Reference)	0.17
Yes	1.51 (1.05, 2.18)		1.53 (1.03, 2.28)		Yes	1.03 (0.75, 1.42)		1.25 (0.90, 1.73)	
**Cerebral palsy at 5**$\frac{1}{2}$ **years**									
No	1.00 (Reference)	<0.001	1.00 (Reference)	0.01	No	1.00 (Reference)	0.02	1.00 (Reference)	0.07
Yes	2.45 (1.42, 4.22)		2.18 (1.28, 3.69)		Yes	1.76 (1.08, 2.85)		1.56 (0.97, 2.52)	

a *Unadjusted ORs weighted to account for differences in the sampling process between gestational age groups*.

b *Wald chi-square p-value*.

c *aORs; 95% confidence interval (CI); adjusted for all variables in the table, GEEs multivariable regression model*.

d *Defined as birth weight less than the 10th centile for gestational age and sex based on French EPOPé intrauterine growth curves (Ego 2016)*.

The odds of posterior crossbite were increased for children with pacifier-sucking habits and children with CP (*p* = 0.07), although not significantly. Posterior crossbite was not associated with sex, duration of intubation, oral stimulation in a NICU, or thumb-sucking habits.

The results of the analyses of complete cases and children with missing data for NNSHs provided aORs very similar to those of children with complete data for NNSHs (Supplementary Tables S2, S3).

## Discussion

In this study, 8% of children had a high arched-palate and 15% a posterior crossbite at the age of 5. In 30% of cases of high-arched palate, these anomalies were associated with each other. High-arched palate was frequent in children with thumb-sucking habits and those with CP. Posterior crossbite was frequent in children with pacifier-sucking.

All data were collected prospectively, which includes pacifier and thumb-sucking habits at the age of 2, thus avoiding recall bias. The study population-based design and the large sample size provided reasonable precision in estimating frequencies and associations.

### Limitations

The main limitation of the study was missing data, mostly due to lost to follow-up at the ages of 2 and 5; multiple imputation was used to reduce the potential selection bias arising from this situation. Children with missing data for NNSHs were often born to mothers born outside of France and with a low socioeconomic background and were infrequently breastfed at discharge; thus, the association between breastfeeding and both outcomes might be underestimated.

In our study, both outcomes were assessed by the pediatricians. High-arched palate is present in many syndromic diseases (38, 39), and pediatricians are familiar with this clinical feature. Moreover, pediatricians were assisted by drawings and a photo chart for better consistency. However, pediatricians are not specialist in this area, even when specifically trained, we assumed that the more severe situations were more likely to be detected, situations that would most probably need orthodontic treatment; therefore, the frequency of high-arched palate is likely underestimated. The definition of posterior crossbite was more precise than that of high-arched palate, but its assessment was probably more unusual for examiners. Thus, even with the guidance chart, the pediatricians may have more difficulties with assessment, possibly leading to non-differential misclassification, which would imply an underestimation of posterior crossbite frequency.

### Frequency of High-Arched Palate and Posterior Crossbite

Comparing the frequency of high-arched palate, we observed with that in the literature is difficult because of no standard definition (40). The previous studies among preterm or low-birth-weight infants have been conducted in the United States (26, 27), Brazil (28), and Japan (29), but included only small samples (37 to 74 children). However, the results of our study were consistent with those from the previous EPIPAGE study (13).

The frequency of posterior crossbite we observed was slightly lower than that observed in Finland (17% of children born preterm under the age of 6) (11) and the United States (17% of low-birth-weight children aged 2–5 and 22% of children aged 3–5) (26, 27). However, such frequencies do not vary much from the frequencies observed in the general population (up to 25%) (16, 24, 41).

### High-Arched Palate and Posterior Crossbite

We studied high-arched palate and posterior crossbite and associated risk factors in the same cohort of children born very preterm because both are related to possible problems in chewing, phonation, mouth breathing, and facial asymmetry. Both are the disorders of the transverse plane and are therefore linked, but they are neither superimposable nor embedded. High-arched palate is a more severe anomaly because growth in height occurs at the expense of width. However, posterior crossbite reflects a discrepancy in the relation between the upper maxillary arch and the lower mandibular arch. If both upper and lower arches are narrow, there is no posterior crossbite, but the situation may need treatment. If only one upper tooth is oriented lingually, there may be a posterior crossbite, but with less need for treatment.

### Non-Nutritive Sucking Habits and Maxillofacial Growth Anomalies

Prolonged NNSHs have been associated with maxillofacial growth anomalies in children, including high-arched palate and posterior crossbite (14–16, 42–45). The habit of a thumb or a pacifier in the mouth accustoms the tongue to be in a low position where it cannot exert pressure on the palate, which potentially results in a high-arched palate. In contrast, low tongue position puts more constant pressure on the lower arch, thus enhancing its growth in the transverse direction and creating a growth discrepancy between the two arches and potential posterior crossbite (46). The evidence suggests that at least 2 years of pacifier use are necessary for substantial alteration in palate morphology (47). Preterm children seem more likely to have NNSHs than full-term children (69 vs. 51% at the age of 2) (16, 21).

This study demonstrated that thumb-sucking at the age of 2 was associated with high-arched palate at the age of 5 and pacifier-sucking with posterior crossbite. The vertical pressure of the thumb on the palate may be stronger than the pressure of the pacifier, thus leading to a deep palate. The studies of the general population in the United States and Europe found similar associations (16, 45, 48).

### Neonatal Factors and Maxillofacial Growth Anomalies

In our study, frequency of high-arched palate was high for children born at low gestational age. In addition to an immature sucking pattern, infants born very preterm are exposed to invasive neonatal care such as mechanical ventilation by a naso- or orotracheal tube. Orotracheal intubation seems to be associated with increased risk of high-arched palate or palatal groove (26, 28, 29). In our study, prolonged intubation was not associated with high-arched palate, perhaps because intubation is mainly nasal in France.

Neonatal units promote developmental practices (including oral stimulation and breastfeeding), which encourage sucking and feeding skills necessary for survival and better development of infants born very preterm. We expected such practices to play a protective role preventing the development of maxillofacial growth anomalies; however, oral stimulation was not associated with the anomalies in our study. Oral stimulation may include heterogeneous intraoral practices (sometimes with the use of pacifiers) and less “intrusive” practices (e.g., chin-only stimulation), which might not have the same impact on palatal growth.

Breastfeeding was associated with less NNSHs in children born very preterm (21). The recent studies of preterm children and the general population in Brazil concluded shorter duration of breastfeeding associated with increased risk of malocclusions (including posterior crossbite) at the age of 5 (25, 49). However, in one such study (25), the association between breastfeeding and posterior crossbite was observed only when it was not adjusted for NNSHs. Once adjusted for NNSHs, the association disappeared, as in our study. Because breastfeeding and NNSHs are closely related, in our case, we could not clearly distinguish the role that breastfeeding plays in the development of high-arched palate and posterior crossbite.

### Cerebral Palsy and Maxillofacial Growth Anomalies

Cerebral palsy was assessed at the age of 5, but it reflects neurological disturbances that occur in the infant brain much earlier in life. Our study confirmed that children with CP are particularly at risk of high-arched palate. CP involves a limitation of oro-facial motor skills (50, 51), so the lingual pressure on the palate may be insufficient, possibly altering its growth. This result endorses close monitoring of maxillofacial growth in infants with CP born preterm. It also generates new research questions about specific care and future treatments that could be used for this spectrum of disorders.

### Maxillofacial Growth Anomalies and Orthodontic Treatment

Early interceptive orthodontic treatment aims to reduce the severity of maxillofacial growth anomalies or prevent a situation from becoming more severe. Interceptive treatment should be used at an early age (between 4–9 years old), its goal being to reduce the potential functional and aesthetic consequences of these anomalies over time. For example, in the case of a growth deficit in the upper maxillary arch, palatal expansion appliances are often used to help to stimulate the growth of the maxilla transversely (52). In the case of a posterior crossbite, removable expansion plates are often used to help to guide the growth of the upper maxillary alveolar processes outside the mandibular arch and thus reduce the risk of persistent malocclusion in the adult dentition (3). Another option is the use of a fixed appliance (Quand-helix) (53) that can help rotation of molars or create expansion at the premolar and canine levels (54).

### A Key Diagnosis for Pediatricians

Upper airway dimensions and craniofacial morphology are closely related (55). High-arched palate is often associated with mouth breathing and may even be a risk factor for sleep disorders breathing. (8, 56, 57). The recent research has assessed different orthodontic appliances (such as rapid maxillary expansion) as treatment modalities in pediatric obstructive sleep apnea (58). Even if evidence is still low to conclude, interdisciplinary collaboration between pediatricians and dentists such as sharing of early diagnoses could make it possible to prevent and treat these various disorders at the early stages. Moreover, high-arched palate could be a call sign for sleep-disordered breathing in child examination.

## Conclusions

In this study among children born very preterm, we found high-arched palate associated with thumb-sucking habits and CP and posterior crossbite mainly associated with pacifier-sucking habits. The high frequency of both disorders justifies particular attention owing to potential longer-term consequences, as shown in other studies. The oral health of infants born very preterm should be regularly monitored throughout early childhood to identify and address these problems at the young age, especially when children have neuromotor dysfunctions or prolonged NNSHs.

Standardized definition of high-arched palate and other types of palatal alteration, which includes grooving, should be developed. Such definitions would help to improve the design of future clinical and observational studies and understanding the mechanisms associated with the occurrence of maxillofacial growth anomalies in general population and in children born preterm.

## Data Availability Statement

The raw data supporting the conclusions of this article will be made available by the authors, without undue reservation.

## Ethics Statement

The studies involving human participants were reviewed and approved by French Data Protection Authority (Commission Nationale de l'informatique et des Libertés, No. 911009) and the two relevant Ethics Committees [the Consultative Committee on the Treatment of Information on Personal Health Data for Research Purposes (CCTIRS), No. 10.626, and the Committee for the Protection of People Participating in Biomedical Research (CPP), No. SC-2873]. Written informed consent to participate in this study was provided by the participants' legal guardian/next of kin. Written informed consent was obtained from the individual(s), and minor(s)' legal guardian/next of kin, for the publication of any potentially identifiable images or data included in this article.

## Author Contributions

SH conceptualized the study, carried out the analysis, and drafted the initial manuscript. VP, P-YA, and MK conceptualized and designed the study. VB coordinated and supervised the data collection. EL designed the data collection instrument. LM-M and AM supervised the statistical analysis. AG designed the study and supervised the statistical analysis. All authors revised and approved the final manuscript as submitted and agree to be accountable for all aspects of the work.

## Funding

The EPIPAGE-2 Study was supported by the French Institute of Public Health Research/Institute of Public Health and its partners: the French Health Ministry; the National Institute of Health and Medical Research (INSERM); the National Institute of Cancer, and the National Solidarity Fund for Autonomy (CNSA); The National Research Agency through the French EQUIPEX program of investments in the future (Reference ANR-11-EQPX-0038); the PREMUP Foundation; and the Foundation de France (Reference 00050329).

## Epipage-2 Study Group

Alsace: D. Astruc, P. Kuhn, B. Langer, J. Matis (Strasbourg), C. Ramousset; Aquitaine: X. Hernandorena (Bayonne), P. Chabanier, L. Joly-Pedespan (Bordeaux), M.J. Costedoat, A. Leguen; Auvergne: B. Lecomte, D. Lemery, F. Vendittelli (Clermont-Ferrand); Basse-Normandie: G. Beucher, M. Dreyfus, B. Guillois (Caen), Y. Toure; Bourgogne: A. Burguet, S Couvreur, J.B. Gouyon, P. Sagot (Dijon), N. Colas; Bretagne: J. Sizun (Brest), A. Beuchée, P. Pladys, F. Rouget (Rennes), R.P. Dupuy (St-Brieuc), D. Soupre (Vannes), F. Charlot, S. Roudaut; Centre: A. Favreau, E. Saliba (Tours), L. Reboul; Champagne-Ardenne: N. Bednarek, P. Morville (Reims), V. Verrière; Franche-Comté: G. Thiriez (Besançon), C. Balamou; Haute-Normandie: L. Marpeau, S. Marret (Rouen), C. Barbier; Ile-de-France: G. Kayem (Colombes), X. Durrmeyer (Créteil), M. Granier (Evry), M. Ayoubi, A. Baud, B. Carbonne, L. Foix L'Hélias, F. Goffinet, P.H. Jarreau, D. Mitanchez (Paris), P. Boileau (Poissy), L. Cornu, R. Moras; Languedoc-Roussillon: P. Boulot, G. Cambonie, H. Daudé (Montpellier), A. Badessi, N. Tsaoussis; Limousin: A. Bédu, F. Mons (Limoges), C. Bahans; Lorraine: M.H. Binet, J. Fresson, J.M. Hascoët, A. Milton, O. Morel, R. Vieux (Nancy), L. Hilpert; Midi-Pyrénées: C. Alberge, C. Arnaud, C. Vayssière (Toulouse), M. Baron; Nord-Pas-de-Calais: M.L. Charkaluk, V. Pierrat, D. Subtil, P. Truffert (Lille), S. Akowanou, D. Roche; PACA et Corse: C. D'Ercole, C. Gire, U. Simeoni (Marseille), A. Bongain (Nice), M. Deschamps; Pays de Loire: B. Branger (FFRSP), J.C. Rozé, N. Winer (Nantes), V. Rouger, C. Dupont; Picardie: J. Gondry, G. Krim (Amiens), B. Baby; Rhône-Alpes: M. Debeir (Chambéry), O. Claris, J.C. Picaud, S. Rubio-Gurung (Lyon), C. Cans, A. Ego, T. Debillon (Grenoble), H. Patural (Saint-Etienne), A. Rannaud; Guadeloupe: E. Janky, A. Poulichet, J.M. Rosenthal (Point à Pitre), E. Coliné; Guyane: A. Favre (Cayenne), N. Joly; Martinique: S. Châlons (Fort de France), V. Lochelongue; La Réunion: P.Y. Robillard (Saint-Pierre), S. Samperiz, D. Ramful (Saint-Denis).

Inserm UMR 1153: P.Y. Ancel, V. Benhammou, B. Blondel, M. Bonet, A. Brinis, M.L. Charkaluk, A. Coquelin, M. Durox, L. Foix-L'Hélias, F. Goffinet, M. Kaminski, G. Kayem, B. Khoshnood, C. Lebeaux, L. Marchand-Martin, A.S. Morgan, V. Pierrat, J. Rousseau, M.J. Saurel-Cubizolles, D. Sylla, D. Tran, L. Vasante-Annamale, J. Zeitlin.

## Conflict of Interest

The authors declare that the research was conducted in the absence of any commercial or financial relationships that could be construed as a potential conflict of interest.

## Publisher's Note

All claims expressed in this article are solely those of the authors and do not necessarily represent those of their affiliated organizations, or those of the publisher, the editors and the reviewers. Any product that may be evaluated in this article, or claim that may be made by its manufacturer, is not guaranteed or endorsed by the publisher.
