# Supplementary material for: Risk Factors for High-Arched Palate and Posterior Crossbite at the Age of 5 in Children Born Very Preterm: EPIPAGE-2 Cohort Study
Source: Front Pediatr. 2022 Apr 15;10:784911. doi: 10.3389/fped.2022.784911 (PMC9051072; doi:10.3389/fped.2022.784911)
Supplement: Supplementary file 1 [file Table_1.DOCX]

| **Supplementary Table 1**  Multiple imputation variables: model used to predict missing data and percentages of values missing for each variable included in the imputation model (N=2594 survivors at 5½ years with complete data on non-nutritive sucking habits [NNSHs] at 2 years) | | | |  |  |
| --- | --- | --- | --- | --- | --- |
| Variable | Type of variable | Model used to predict missing data | Percentages of values missing | |  |
| High-arched palate | Binary | Logistic regression | 30% | |  |
| Posterior crossbite | Binary | Logistic regression | 36% | |  |
| Maternal country of birth | Binary | Logistic regression | <1% | |  |
| Parents' socioeconomic status^a^ | Categorical (6 categories) | Multinomial regression | 4% | |  |
| Parity | Categorical (3 categories) | Multinomial regression | <1% | |  |
| Type of pregnancy | Binary | No missing data | 0% | |  |
| Sex | Binary | No missing data | 0% | |  |
| Gestational age (weeks) | Categorical (3 categories) | No missing data | 0% | |  |
| Small-for-gestational-age^b^ | Binary | No missing data | 0% | |  |
| Severe neonatal morbidities^c^ | Binary | Logistic regression | 4% | |  |
| Intubation | Categorical (3 categories) | No missing data | <1% | |  |
| Feeding by nasogastric tube at 36 weeks | Binary | Logistic regression | 11% | |  |
| Oral stimulation | Binary | Logistic regression | 5% | |  |
| Breastfeeding at discharge | Binary | Logistic regression | 5% | |  |
| Pacifier-sucking habits at 2 years | Binary | Logistic regression | 0% | |  |
| Thumb-sucking habits at 2 years | Binary | Logistic regression | 0% | |  |
| Cerebral palsy at 5½ years | Binary | Logistic regression | 20% | |  |
| ^a^Defined as the highest occupational status of the mother and father or occupation of mother only if living alone. | | | | | |
| ^b^Defined as birth weight less than the 10th centile for gestational age and sex based on the French EPOPé intrauterine growth curves (Ego 2016). | | | | | |
| ^c^Defined as severe bronchopulmonary dysplasia or necrotizing enterocolitis stage 2-3, severe retinopathy of prematurity stage >3 or any of the following severe cerebral abnormalities on cranial ultrasonography: intraventricular hemorrhage grade III/IV or cystic periventricular leukomalacia. | | | | | |
