# Supplementary material for: Risk Factors for High-Arched Palate and Posterior Crossbite at the Age of 5 in Children Born Very Preterm: EPIPAGE-2 Cohort Study
Source: Front Pediatr. 2022 Apr 15;10:784911. doi: 10.3389/fped.2022.784911 (PMC9051072; doi:10.3389/fped.2022.784911)
Supplement: Supplementary file 2 [file Table_2.DOCX]

| **Supplementary Table 2** | | | | |
| --- | --- | --- | --- | --- |
| Sensitivity analyses: high-arched palate at 5½ years by neonatal characteristics, non-nutritive sucking habits (NNSHs) at 2 years and cerebral palsy at 5½ years; adjusted odds ratios (ORs), multivariable regression models with generalized estimating equations (GEEs), for 1) complete cases and 2) all survivors at 2 years | | | | |
|  | High-arched palate | | | |
|  | *Complete cases* | | *All survivors at 2 years* | |
|  | aOR (95% CI)^a^ | *P^b^* | aOR (95% CI)^c^ | *P^b^* |
|  | **N=1386** |  | **N=3099** |  |
| Sex |  |  |  |  |
| Boys | 1.00 (Reference) | 0.99 | 1.00 (Reference) | 0.98 |
| Girls | 0.99 (0.65, 1.52) |  | 0.99 (0.72, 1.37) |  |
| Gestational age (weeks) |  |  |  |  |
| 24-26 | 1.34 (0.68, 2.65) | 0.26 | 1.28 (0.75, 2.18) | 0.05 |
| 27-29 | 1.56 (0.95, 2.52) |  | 1.62 (1.09, 2.41) |  |
| 30-31 | 1.00 (Reference) |  | 1.00 (Reference) |  |
| Small-for-gestational age^d^ |  |  |  |  |
| No | 1.00 (Reference) | 0.21 | 1.00 (Reference) | 0.26 |
| Yes | 1.31 (0.86, 1.99) |  | 1.21 (0.87, 1.68) |  |
| Intubation |  |  |  |  |
| < 24 hr | 1.00 (Reference) | 0.30 | 1.00 (Reference) | 0.16 |
| 24 hr-28 days | 1.50 (0.88, 1.55) |  | 1.38 (0.84, 1.27) |  |
| > 28 days | 1.18 (0.45, 3.06) |  | 1.52 (0.76, 3.04) |  |
| Oral stimulation |  |  |  |  |
| No | 1.00 (Reference) | 0.44 | 1.00 (Reference) | 0.61 |
| Yes | 1.20 (0.75, 1.92) |  | 0.90 (0.59, 1.36) |  |
| Breastfeeding at discharge |  |  |  |  |
| No | 1.00 (Reference) | 0.01 | 1.00 (Reference) | 0.06 |
| Yes | 0.57 (0.37, 0.89) |  | 0.70 (0.48, 1.02) |  |
| Pacifier-sucking at 2 years |  |  |  |  |
| Non | 1.00 (Reference) | 0.84 | 1.00 (Reference) | 0.39 |
| Yes | 0.93 (0.44, 1.98) |  | 1.17 (0.81, 1.70) |  |
| Thumb-sucking at 2 years |  |  |  |  |
| Non | 1.00 (Reference) | 0.03 | 1.00 (Reference) | 0.01 |
| Yes | 2.00 (1.03, 3.88) |  | 1.55 (1.09, 2.21) |  |
| Cerebral palsy at 5½ years |  |  |  |  |
| No | 1.00 (Reference) | 0.08 | 1.00 (Reference) | <0.01 |
| Yes | 1.78 (0.96, 3.35) |  | 2.08 (1.23, 3.53) |  |
| ^a^aORs; 95% confidence interval (CI); adjusted for all variables in the table, GEEs multivariable regression model. | | | | |
| ^b^Wald chi-square p-value. | | | | |
| ^c^aORs; 95% CI; adjusted for all variables in the table, GEEs multivariable regression model with multiple imputation. | | | | |
| ^d^Defined as birth weight less than the 10th centile for gestational age and sex based on French EPOPé intrauterine growth curves (Ego 2016). | | | | |
