# Supplementary material for: Risk Factors for High-Arched Palate and Posterior Crossbite at the Age of 5 in Children Born Very Preterm: EPIPAGE-2 Cohort Study
Source: Front Pediatr. 2022 Apr 15;10:784911. doi: 10.3389/fped.2022.784911 (PMC9051072; doi:10.3389/fped.2022.784911)
Supplement: Supplementary file 3 [file Table_3.DOCX]

| **Supplementary Table 3** | | | | |
| --- | --- | --- | --- | --- |
| Sensitivity analyses: posterior crossbite at 5½ years by neonatal characteristics, non-nutritive sucking habits (NNSHs) at 2 years and cerebral palsy at 5½ years; adjusted odds ratios (ORs), multivariable regression models with generalized estimating equations (GEEs), for 1) complete cases and 2) all survivors at 2 years | | | | |
|  | Posterior crossbite | | | |
|  | *Complete cases* | | *All survivors at 2 years* | |
|  | aOR (95% CI)^a^ | *P^b^* | aOR (95% CI)^c^ | *P^b^* |
|  | **N=1386** |  | **N=3099** |  |
| Sex |  |  |  |  |
| Boys | 1.00 (Reference) | 0.89 | 1.00 (Reference) | 0.96 |
| Girls | 1.02 (0.75, 1.39) |  | 0.99 (0.78, 1.26) |  |
| Gestational age (weeks) |  |  |  |  |
| 24-26 | 1.26 (0.74, 2.14) | 0.38 | 1.70 (1.14, 2.53) | 0.01 |
| 27-29 | 1.13 (0.78, 1.62) |  | 1.18 (0.85, 1.63) |  |
| 30-31 | 1.00 (Reference) |  | 1.00 (Reference) |  |
| Small-for-gestational age^d^ |  |  |  |  |
| No | 1.00 (Reference) | 0.75 | 1.00 (Reference) | 0.50 |
| Yes | 0.95 (0.70, 1.30) |  | 0.91 (0.68, 1.20) |  |
| Intubation |  |  |  |  |
| < 24 hr | 1.00 (Reference) | 0.05 | 1.00 (Reference) | 0.14 |
| 24 hr-28 days | 0.77 (0.54, 1.11) |  | 0.84 (0.60, 1.16) |  |
| > 28 days | 0.46 (0.20, 1.09) |  | 0.60 (0.30, 1.23) |  |
| Oral stimulation |  |  |  |  |
| No | 1.00 (Reference) | 0.09 | 1.00 (Reference) | 0.09 |
| Yes | 1.35 (0.95, 1.92) |  | 1.30 (0.95, 1.79) |  |
| Breastfeeding at discharge |  |  |  |  |
| No | 1.00 (Reference) | 0.23 | 1.00 (Reference) | 0.14 |
| Yes | 0.82 (0.60, 1.12) |  | 0.81 (0.61, 1.07) |  |
| Pacifier-sucking at 2 years |  |  |  |  |
| Non | 1.00 (Reference) | 0.03 | 1.00 (Reference) | <0.001 |
| Yes | 1.68 (1.03, 2.73) |  | 1.72 (1.31, 2.25) |  |
| Thumb-sucking at 2 years |  |  |  |  |
| Non | 1.00 (Reference) | 0.47 | 1.00 (Reference) | 0.27 |
| Yes | 1.21 (0.71, 2.09) |  | 1.19 (0.87, 1.63) |  |
| Cerebral palsy at 5½ years |  |  |  |  |
| No | 1.00 (Reference) | 0.09 | 1.00 (Reference) | 0.17 |
| Yes | 1.69 (0.91, 3.13) |  | 1.45 (0.84, 2.49) |  |
| ^a^aORs; 95% confidence interval (CI); adjusted for all variables in the table, GEEs multivariable regression model. | | | | |
| ^b^Wald chi-square p-value. | | | | |
| ^c^aORs; 95% CI; adjusted for all variables in the table, GEEs multivariable regression model with multiple imputation. | | | | |
| ^d^Defined as birth weight less than the 10th centile for gestational age and sex based on French EPOPé intrauterine growth curves (Ego 2016). | | | | |
