# Supplementary material for: Risk Factors for High-Arched Palate and Posterior Crossbite at the Age of 5 in Children Born Very Preterm: EPIPAGE-2 Cohort Study
Source: Front Pediatr. 2022 Apr 15;10:784911. doi: 10.3389/fped.2022.784911 (PMC9051072; doi:10.3389/fped.2022.784911)

Supplementary Figure 1

1. Does X have a high-arched palate?

a. Normal palate (shallow)

☐

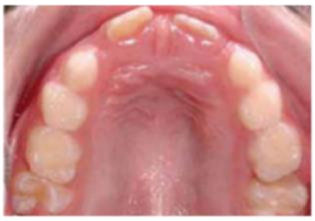

b. High-arched palate (deep and narrow)

☐

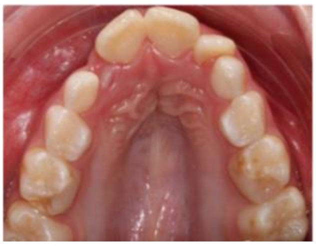

Normal occlusion
(the upper dental arch circumscribes the lower arch)

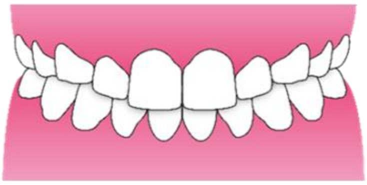

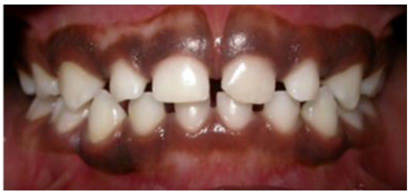

2. Does X have an anterior crossbite? **Yes** ☐ **No** ☐

Anterior crossbite
(at least 2 lower incisors in front of the upper incisors)

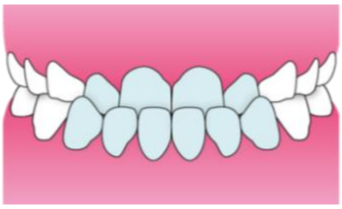

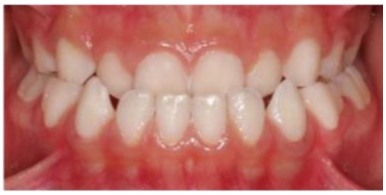

3. Does X have a posterior crossbite? **Yes** ☐ **No** ☐

If yes,

a. **Unilateral posterior crossbite**
(at least 1 lower tooth on the outside, even just a tip, on one side only) ☐

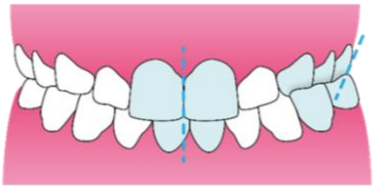

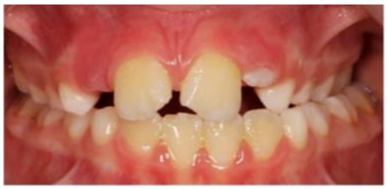

b. **Bilateral posterior crossbite**
(at least 1 lower tooth on the outside, even just a tip, on both sides) ☐

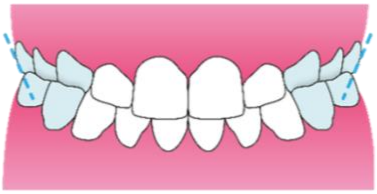

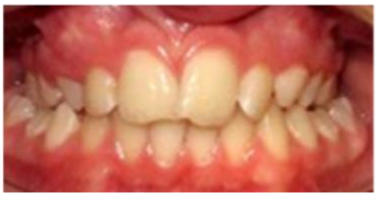

Supplement: Supplementary file 4 [file Data_Sheet_1.PDF]
